# Pregnancy-Associated Mortality Due to Homicide, Suicide, and Drug Overdose

**DOI:** 10.1001/jamanetworkopen.2024.59342

**Published:** 2025-02-11

**Authors:** Maeve E. Wallace, Jaquelyn L. Jahn

**Affiliations:** 1 Department of Health Promotion Sciences, Mel and Enid Zuckerman College of Public Health, University of Arizona, Tucson; 2 Department of Social, Behavioral, and Population Sciences, Celia Scott Weatherhead School of Public Health and Tropical Medicine, Tulane University, New Orleans, Louisiana; 3 The Ubuntu Center on Racism, Global Movements, and Population Health Equity, Dornsife School of Public Health, Drexel University, Philadelphia, Pennsylvania; 4 Department of Epidemiology and Biostatistics, Dornsife School of Public Health, Drexel University, Philadelphia, Pennsylvania

## Abstract

**Importance:**

Despite growing national concern about high and increasing rates of pregnancy-associated mortality due to homicide, suicide, and drug overdose, state-level incidence has previously not been available.

**Objective:**

To identify cases of pregnancy-associated homicide, suicide, drug overdose, and deaths involving firearms in the US from calendar year 2018 to 2022 and estimate 5-year proportionate mortality and mortality ratios per 100 000 live births by state and cause of death.

**Design, Setting, and Participants:**

This cross-sectional study is a population-based analysis of the 2018-2022 restricted-use mortality files provided by the National Center for Health Statistics. These data include all deaths occurring in the US, with geographic identifiers for state of residence. All records in which the decedent was female aged 10 to 44 years and pregnant at the time of death or up to 1 year earlier were included in the analysis. Data were analyzed from July 1 to December 1, 2024.

**Main Outcomes and Measures:**

*International Statistical Classification of Diseases and Related Health Problems, Tenth Revision* codes for underlying cause of death were used to identify cases of homicide, suicide, drug overdose, and deaths involving firearms occurring in each state from 2018 to 2022. Proportionate mortality was estimated as the count of cases divided by the total count of deaths of pregnant and postpartum women in each state. Cause-specific mortality ratios were estimated as the count of cases divided by the total count of live births in each state from 2018 to 2022.

**Results:**

Nationally, there were 10 715 deaths of people who were pregnant or within 1 year post partum from 2018 to 2022, including 837 homicides, 579 suicides, 2083 drug overdoses, and 851 that involved firearms. Proportionate mortality and mortality ratios for homicide, suicide, and drug overdose varied across the US. Of states with more than 9 cases, pregnancy-associated homicide mortality was highest in Mississippi (12.86 per 100 000 live births), pregnancy-associated suicide mortality was highest in Montana (21.55 deaths per 100 000 live births), and pregnancy-associated drug overdose was highest in Delaware (36.03 deaths per 100 000 live births). Firearms accounted for as many as 15.56% of pregnancy-associated deaths in Colorado, and pregnancy-associated firearm mortality was highest in Mississippi (13.42 deaths per 100 000 live births).

**Conclusions and Relevance:**

The information in this study may provide relevant guidance for state and local intervention strategies to advance the health, safety, and well-being of women during pregnancy and beyond.

## Key Points

**Question:**

Does mortality from pregnancy-associated homicide, suicide, drug overdose, and firearms vary across the US?

**Findings:**

This cross-sectional analysis of the first 5 years of nationally available data on pregnancy-associated mortality in 10 715 females found substantial state variation in the proportion of deaths during pregnancy and up to 1 year post partum that are associated with homicide, suicide, and drug overdose and in cause-specific mortality ratios (deaths per 100 000 live births). Firearms are a major factor in the violent deaths of pregnant and postpartum women in many states.

**Meaning:**

The findings of this study suggest that state-specific policy and programmatic intervention strategies may be needed to ensure all women experience safe and healthy pregnancies.

## Teaser

This cross-sectional study examines pregnancy-associated homicide, suicide, drug overdose, and death by firearms from 2018 to 2022 throughout the US.

## Introduction

Death during pregnancy and up to 1 year post partum (pregnancy-associated mortality) is frequent in the US,^1,2^ including pregnancy-related deaths (those related to or aggravated by the pregnancy or its management) and unintentional or incidental causes of death. Recent research has shed new light on the role of homicide, suicide, and drug overdose during pregnancy and the postpartum period as major factors in pregnancy-associated mortality, raising concern about their high frequency and recent increases nationally.^2,3,4^

Estimates of pregnancy-associated deaths due to violence (homicide and suicide) and drug overdose have been reported in some subnational jurisdictions,^5,6,7^ but a more comprehensive description of state-level prevalences has previously not been possible. Data use agreements prohibit the estimation of rates based on a case count of less than 10 for both reliability and confidentiality concerns.^8^ Given the relative rarity of these deaths, estimating annual state-level rates in these outcomes is prohibited in most cases, especially in more sparsely populated states. With national implementation of enhanced pregnancy surveillance on US death certificates recently complete, availability of state-specific data on pregnancy-associated mortality will continue to increase as more years of data are included.

The purpose of this analysis is to describe pregnancy-associated homicide, suicide, and drug overdose at the state-level to guide and support more localized efforts to promote maternal health and safety. We used data from 2018, the first year of fully national reporting, through the most recently available year (2022) to estimate 5-year mortality ratios by state for pregnancy-associated deaths due to violence, firearms, and drug overdose.

## Methods

We identified all cases of pregnancy-associated mortality in the 2018-2022 nationally restricted use mortality files (death certificates with a geographic identifier for state of residence) as records where the pregnancy checkbox indicated the decedent was pregnant or within 1 year from the end of pregnancy at the time of death. Given concern about misclassification arising from the pregnancy checkbox for decedents at more advanced ages,^9^ we limited the data to females between the ages of 10 and 44 years. Cases of homicide and suicide were those with a manner of death indicating as such and/or an *International Statistical Classification of Diseases and Related Health Problems, Tenth Revision* (*ICD-10*) code for underlying cause of death in X85-Y09 for homicide and X60-X84, Y87.0, or U03 for suicide. Cases of drug overdose were identified using the *ICD-10* coding scheme proposed by Margerison et al,^2^ which includes only unintentional overdose or cases where intent is unknown. This analysis of deidentified vital records data was ruled exempt by the Tulane University Institutional Review Board, and this report follows the Strengthening the Reporting of Observational Studies in Epidemiology (STROBE) reporting guideline. Data were analyzed from July 1 to December 1, 2024.

### Statistical Analysis

We summed case counts over the 5-year period in every state and estimated proportionate mortality by cause of death (cause-specific count of deaths divided by total deaths). To estimate mortality ratios per 100 000 live births, we divided cause-specific counts of death by the sum of live births in people aged 10 to 44 years in each state over the same time using the 2018-2022 restricted-use natality files (birth certificates with a geographic identifier for state of residence). In addition, we estimated the proportionate mortality due to firearms (*ICD-10* codes X93-X95 for homicide and X72-X74 for suicide) and the pregnancy-associated firearm mortality ratio as deaths per 100 000 live births. We suppressed data for any state where the case count was greater than 0 and less than 10 to preserve anonymity. Analysis was performed with SAS, version 9.4 (SAS Institute Inc).

## Results

Nationally, there were 10 715 deaths of people who were pregnant or within 1 year post partum from 2018 to 2022, including 837 homicides, 579 suicides, 2083 drug overdoses, and 851 that involved firearms. Table 1 lists the total number of pregnancy-associated deaths due to any cause and the number and percentage of these deaths that were due to homicide, suicide, and drug overdose in each state where data suppression rules allow (ie, where there were 0 or >9 cases). Nationally, 51.4% of homicides, 31.8% of suicides, and 34.4% of overdoses included in this study happened during pregnancy; the remaining were within the first year post partum.

**Table 1.** State Pregnancy-Associated Mortality Case Counts and Proportionate Mortality by Cause, 2018-2022^a^

State	All pregnancy-associated deaths, No.	Pregnancy-associated deaths due to homicide, suicide, or drug overdose, No. (%)	No. (%) of pregnancy-associated deaths		
			Homicide	Suicide	Drug overdose
Alabama	273	56 (20.5)	18 (6.6)	13 (4.8)	25 (9.2)
Alaska	23	NR	NR	NR	NR
Arizona	258	70 (27.1)	20 (7.8)	12 (4.7)	38 (14.7)
Arkansas	138	28 (20.3)	13 (9.4)	NR	NR
California	607	146 (24.1)	38 (6.3)	26 (4.3)	82 (13.5)
Colorado	180	88 (48.9)	18 (10.0)	35 (19.4)	35 (19.4)
Connecticut	78	24 (30.8)	NR	NR	18 (23.1)
Delaware	39	23 (59.0)	NR	NR	19 (48.7)
District of Columbia	30	NR	NR	0	NR
Florida	580	189 (32.6)	53 (9.1)	29 (5.0)	107 (18.4)
Georgia	568	177 (31.2)	69 (12.1)	32 (5.6)	76 (13.)
Hawaii	31	11 (35.5)	NR	NR	NR
Idaho	59	13 (22.0)	NR	NR	NR
Illinois	284	114 (40.1)	28 (9.9)	20 (7.0)	66 (23.2)
Indiana	277	94 (33.9)	17 (6.1)	13 (4.7)	64 (23.1)
Iowa	82	18 (22.0)	NR	NR	NR
Kansas	85	20 (23.5)	NR	NR	NR
Kentucky	213	72 (33.8)	14 (6.6)	NR	50 (23.5)
Louisiana	246	79 (32.1)	27 (11.0)	NR	47 (19.1)
Maine	20	NR	0	0	NR
Maryland	187	70 (37.4)	18 (9.6)	NR	45 (24.1)
Massachusetts	119	41 (34.5)	NR	NR	33 (27.7)
Michigan	306	106 (34.6)	29 (9.5)	15 (4.9)	62 (20.3)
Minnesota	129	59 (45.7)	NR	11 (8.5)	39 (30.2)
Mississippi	186	51 (27.4)	23 (12.4)	NR	23 (12.4)
Missouri	338	151 (44.7)	41 (12.1)	19 (5.6)	91 (26.9)
Montana	56	21 (37.5)	NR	12 (21.4)	NR
Nebraska	67	14 (20.9)	NR	NR	NR
Nevada	58	17 (29.3)	NR	NR	11 (19.0)
New Hampshire	36	21 (58.3)	0	NR	17 (47.2)
New Jersey	252	59 (23.4)	10 (4.0)	NR	44 (17.5)
New Mexico	89	29 (32.6)	NR	NR	16 (18.0)
New York	605	218 (36.0)	32 (5.3)	35 (5.8)	151 (25.0)
North Carolina	575	232 (40.3)	52 (9.0)	24 (4.2)	156 (27.1)
North Dakota	32	12 (37.5)	NR	NR	NR
Ohio	494	233 (47.2)	37 (7.5)	23 (4.7)	173 (35.0)
Oklahoma	110	18 (16.4)	NR	NR	NR
Oregon	102	34 (33.3)	NR	NR	21 (20.6)
Pennsylvania	386	176 (45.6)	33 (8.5)	21 (5.4)	122 (31.6)
Rhode Island	15	NR	0	0	NR
South Carolina	235	78 (33.2)	32 (13.6)	NR	38 (16.2)
South Dakota	53	17 (32.1)	NR	NR	NR
Tennessee	322	92 (28.6)	20 (6.2)	NR	67 (20.8)
Texas	1001	204 (20.4)	72 (7.2)	49 (4.9)	83 (8.3)
Utah	89	26 (29.21)	NR	13 (14.6)	10 (11.2)
Vermont	NR	NR	NR	NR	NR
Virginia	339	109 (32.2)	23 (6.8)	17 (5.0)	69 (20.4)
Washington	179	54 (30.2)	NR	15 (8.4)	30 (16.8)
West Virginia	73	28 (38.4)	NR	NR	20 (27.4)
Wisconsin	143	65 (45.5)	15 (10.5)	10 (7.0)	40 (28.0)
Wyoming	22	9 (40.9)	NR	NR	NR

Abbreviation: NR, not reported.

^a^ Counts greater than 0 and less than 10 are not reported to preserve anonymity.

The Figure maps state-level pregnancy-associated mortality ratios (deaths per 100 000 live births) for homicide, suicide, and drug overdose (eTable in Supplement 1). The count of pregnancy-associated homicides during this period was highest in Texas (72 [3.82 per 100 000 live births]), although the rate was highest in Mississippi (23 [12.86 per 100 000 live births]). The number of pregnancy-associated suicides was also highest in Texas (49 [2.6 per 100 000 live births]), with the rates highest in Montana (12 [21.55 per 100 000 live births]). For pregnancy-associated drug overdose deaths, counts were highest in Ohio (173 [26.38 per 100 000 live births]), and rates were highest in Delaware (19 [36.03 per 100 000 live births]).

**Figure.**
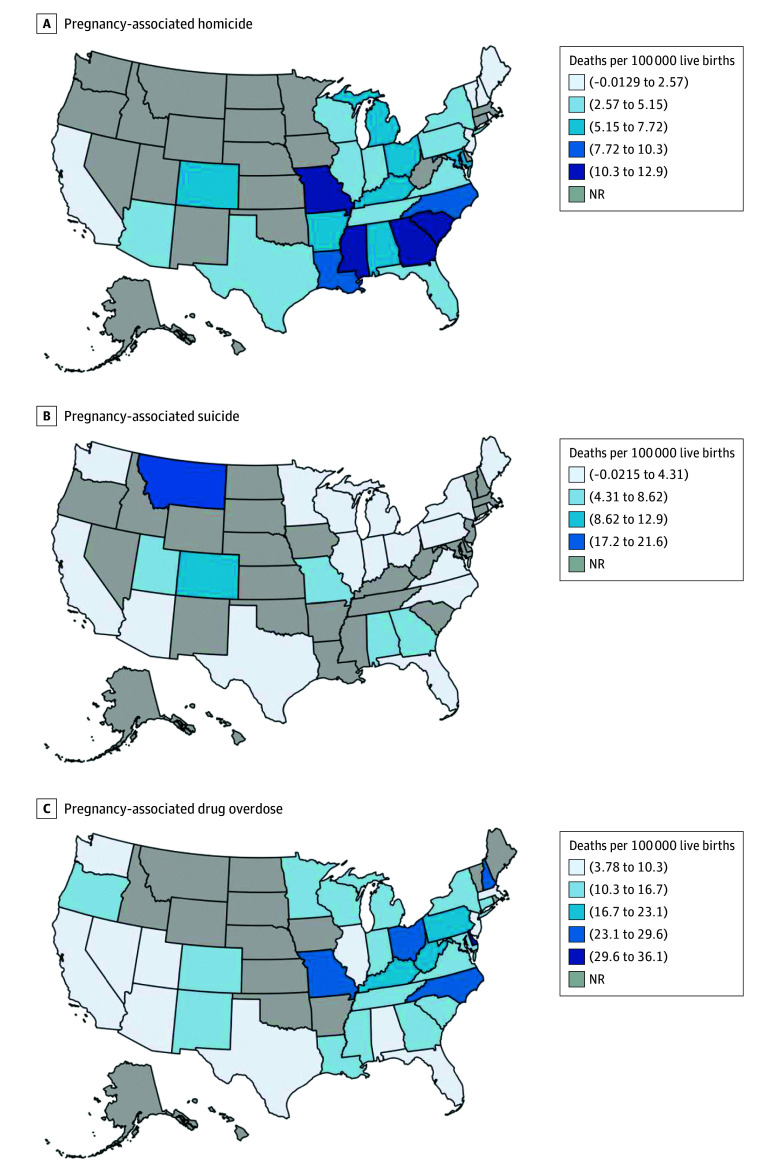
State Pregnancy-Associated Mortality Ratios (Deaths Per 100 000 Live Births), 2018-2022 Pregnancy-associated homicide (A), suicide (B), and drug overdose (C) throughout the US. Cells with counts greater than 0 and less than 10 are not reported to preserve anonymity. NR indicates not reported.

Firearms were by far the most common mechanism of injury in pregnancy-associated homicide, involved in 76% of the cases. Firearms were used in 37% of pregnancy-associated suicides—the second most common mechanism of injury behind hanging and suffocation at 40%. Table 2 depicts the prevalence of firearm involvement by state. In Colorado, 15.56% of pregnancy-associated deaths involved a firearm (28 [8.98 deaths per 100 000 live births]). Mississippi had the highest rate of pregnancy-associated mortality involving a firearm (24 [13.42 per 100 000 live births]). However, there were also 3 states with no pregnancy-associated deaths that were recorded as involving a firearm during this period (Maine, Rhode Island, and Vermont); these states, as well as New Hampshire, also had no recorded pregnancy-associated homicides included in this study.

**Table 2.** State Pregnancy-Associated Mortality Involving Firearms, Proportionate Mortality, and Mortality Ratios (Deaths Per 100 000 Live Births), 2018-2022^a^

State	Pregnancy-associated death involving firearms					
	Total firearm deaths		Homicide		Suicide	
	No. (%)^b^	Firearm mortality ratio	No. (% of pregnancy-associated deaths)	Firearm homicide mortality ratio	No. (% of pregnancy-associated deaths)	Firearm suicide mortality ratio
Alabama	21 (7.7)	7.24	15 (5.5)	5.17	NR	NR
Alaska	NR	NR	NR	NR	NR	NR
Arizona	22 (8.5)	5.60	14 (5.4)	3.56	NR	NR
Arkansas	14 (10.1)	7.77	12 (8.7)	6.66	NR	NR
California	30 (4.9)	1.39	24 (4.0)	1.12	NR	NR
Colorado	28 (15.6)	8.98	11 (6.1)	3.53	17 (9.4)	5.45
Connecticut	NR	NR	NR	NR	0	0
Delaware	NR	NR	NR	NR	0	0
District of Columbia	NR	NR	NR	NR	0	0
Florida	54 (9.3)	4.96	43 (7.4)	3.95	11 (1.9)	1.01
Georgia	70 (12.3)	11.22	53 (9.3)	8.50	17 (3.0)	2.73
Hawaii	NR	NR	NR	NR	0	0
Idaho	NR	NR	0	0	NR	NR
Illinois	27 (9.5)	3.99	22 (7.7)	3.25	NR	NR
Indiana	19 (6.9)	4.75	15 (5.4)	3.75	NR	NR
Iowa	NR	NR	NR	NR	NR	NR
Kansas	NR	NR	NR	NR	NR	NR
Kentucky	18 (8.5)	6.85	13 (6.1)	4.95	NR	NR
Louisiana	24 (9.8)	8.29	22 (8.9)	7.60	NR	NR
Maine	0	0	0	0	0	0
Maryland	15 (8.0)	4.34	14 (7.5)	4.05	NR	NR
Massachusetts	NR	NR	NR	NR	0	0
Michigan	35 (11.4)	6.63	26 (8.5)	4.92	NR	NR
Minnesota	10 (7.8)	3.08	NR	NR	NR	NR
Mississippi	24 (12.9)	13.42	20 (10.8)	11.19	NR	NR
Missouri	45 (13.3)	12.76	35 (10.4)	9.92	10 (2.3)	2.84
Montana	NR	NR	NR	NR	NR	NR
Nebraska	NR	NR	NR	NR	0	0
Nevada	NR	NR	NR	NR	NR	NR
New Hampshire	NR	NR	NR	0	NR	NR
New Jersey	NR	NR	0	NR	0	0
New Mexico	NR	NR	NR	NR	NR	NR
New York	19 (3.14)	1.78	14 (2.3)	1.31	NR	NR
North Carolina	49 (8.5)	8.23	43(7.5)	7.22	NR	NR
Ohio	41 (8.3)	6.25	33 (6.7)	5.03	NR	NR
Oklahoma	NR	NR	NR	NR	NR	NR
Oregon	NR	NR	NR	NR	NR	NR
Pennsylvania	22 (5.7)	3.32	NR	3.02	NR	NR
Rhode Island	0	0	0	0	0	0
South Carolina	31 (13.2)	10.92	28 (11.9)	9.86	NR	NR
South Dakota	NR	NR	NR	NR	NR	NR
Tennessee	20 (6.2)	4.96	17 (5.3)	4.22	NR	NR
Texas	69 (6.9)	3.66	50 (5.0)	2.65	19 (1.9)	1.01
Utah	NR	NR	NR	NR	NR	NR
Vermont	0	0	NR	0	0	0
Virginia	31 (9.1)	6.43	18 (5.3)	3.73	13 (3.8)	2.70
Washington	12 (6.7)	2.86	NR	NR	NR	NR
West Virginia	NR	NR	12 (8.4)	NR	NR	NR
Wisconsin	16 (11.2)	5.17	NR	3.88	NR	NR
Wyoming	NR	NR	NR	NR	0	0

Abbreviation: NR, not reported.

^a^ Counts greater than 0 and less than 10 are not reported to preserve anonymity.

^b^ Denominators for these percentage values can be found in Table 1.

## Discussion

Beginning in 2018, all 50 states and the District of Columbia had completed implementation of the pregnancy checkbox on death records, allowing the certifier to mark the decedent as pregnant or within 1 year from the end of pregnancy at the time of death. Following this milestone, the Centers for Disease Control and Prevention resumed national reporting of maternal mortality ratios, which have been increasing in the years since.^1,9^ Simultaneously, researchers have been using these data to highlight co-occurring trends in pregnancy-associated death more broadly, noting national increases in homicide, suicide, and drug overdose during pregnancy and the postpartum period.^2,3,4,10^ While many of these causes of death among pregnant and postpartum people are more prevalent than obstetric causes, they remain underemphasized in preventive efforts.^11^

We observed substantial variation in pregnancy-associated mortality across the US, implicating the influence of state contexts and policies on population health.^12,13,14,15^ There was wide variation across states in the rates of pregnancy-associated homicides, suicides, and drug overdose mortality, in addition to important state-level heterogeneity in the role of firearms in these deaths. The past decade has witnessed increasing polarization of public policies across states with differing political climates and economic circumstances. This has resulted in measurable changes in health outcomes between state populations and expanding geographic difference life expectancy.^16^ Such policies shape the structural and macrosocial contexts that differ notably between states and that are increasingly understood as root causes of maternal health outcomes and inequities.^17^

### Limitations

Our study is limited by the nature of the administrative data source used herein. These data do not contain detailed contextual information that would allow for better characterization of the circumstances surrounding cases of pregnancy-associated mortality (eg, the role of intimate partner violence, substance use, and mental or physical comorbidities). Second, we only included cases where pregnancy status was known, and given the large proportion of death records among women of reproductive age with pregnancy status marked “unknown” (46.6%), all counts and rates reported in our tables are conservative underestimates of the true magnitude of pregnancy-associated mortality in each state. In addition, given the small case counts within states, we were unable to further stratify our estimates by potentially important characteristics, such as race and ethnicity or timing of death relative to pregnancy. We note that nationally, 51.4% of homicides, 31.8% of suicides, and 34.4% of overdoses during the study timeframe happened during pregnancy, while the remaining were within the first year post partum.

## Conclusions

In this cross-sectional study of pregnancy-associated mortality in the US, we found variation in causes of death and their magnitude across states. With recent federal calls to establish state mortality review boards for both maternal mortality^18^ and violent maternal death,^19^ an evidence base is needed to inform their policy recommendations and guide policy-level interventions. This evidence base includes epidemiologic work that identifies cause-specific pregnancy-associated mortality rates to inform state and national intervention strategies. Moreover, future work must attend to the structural and policy-level causes of differences in rates across states so that women in every state have equal opportunity to experience and survive a healthy pregnancy.

## Notes

Supplement 1. **eTable.** State Pregnancy-Associated Mortality Ratios (Deaths per 100,000 Live Births) for Homicide, Suicide, and Drug Overdose, 2018-2022

Supplement 2.
Data Sharing Statement
