# Supplementary material for: Pregnancy-Associated Mortality Due to Homicide, Suicide, and Drug Overdose
Source: JAMA Netw Open. 2025 Feb 11;8(2):e2459342. doi: 10.1001/jamanetworkopen.2024.59342 (PMC11815526; doi:10.1001/jamanetworkopen.2024.59342)
Supplement: Supplement 1. — eTable. State Pregnancy-Associated Mortality Ratios (Deaths per 100,000 Live Births) for Homicide, Suicide, and Drug Overdose, 2018-2022 [file jamanetwopen-e2459342-s001.pdf]

Supplementary Online Content

Wallace ME, Jahn JL. Pregnancy-associated mortality due to homicide, suicide, and drug overdose. *JAMA Netw Open*. 2025;8(2):e2459342. doi:10.1001/jamanetworkopen.2024.59342

eTable. State Pregnancy-Associated Mortality Ratios (Deaths per 100,000 Live Births) for Homicide, Suicide, and Drug Overdose, 2018-2022

This supplementary material has been provided by the authors to give readers additional information about their work.

© 2025 Wallace ME et al. *JAMA Network Open.*

eTable. State pregnancy-associated mortality ratios (deaths per 100,000 live births) for homicide, suicide, and drug overdose, 2018-2022.[a]

	Pregnancy-associated mortality ratios		
**State**	**Homicide**	**Suicide**	**Drug overdose**
AK	NR	NR	NR
AL	6.21	4.48	8.62
AR	7.22	NR	NR
AZ	5.09	3.06	9.68
CA	1.77	1.21	3.81
CO	5.77	11.23	11.23
CT	NR	NR	10.41
DE	NR	NR	36.03
FL	4.87	2.66	9.83
GA	11.06	5.13	12.18
HI	NR	NR	NR
IA	NR	NR	NR
ID	NR	NR	NR
IL	4.14	2.95	9.75
IN	4.25	3.25	15.99
KS	NR	NR	NR
KY	5.33	NR	19.02
LA	9.33	NR	16.23
MA	NR	NR	9.67
MD	5.21	NR	13.02
ME	0	0	NR
MI	5.49	2.84	11.74
MN	NR	3.39	12.02
MO	11.63	5.39	25.80
MS	12.86	NR	12.86
MT	NR	21.55	NR
NC	8.74	4.03	26.21
ND	NR	NR	NR
NE	NR	NR	NR
NH	0	NR	28.25
NJ	2.00	NR	8.78
NM	NR	NR	14.45
NV	NR	NR	6.44
NY	2.99	3.27	14.11
OH	5.64	3.51	26.38
OK	NR	NR	NR
OR	NR	NR	10.31
PA	4.98	3.17	18.43
RI	0	0	NR

© 2025 Wallace ME et al. *JAMA Network Open.*

SC	11.27	NR	13.38
SD	NR	NR	NR
TN	4.96	NR	16.61
TX	3.82	2.60	4.41
UT	NR	5.61	4.31
VA	4.77	3.53	14.32
VT	0	NR	NR
WA	NR	3.57	7.14
WI	4.85	3.23	12.93
WV	NR	NR	22.81
WY	NR	NR	NR

[a] NR is not reported. Ratios based on numerator counts of >0 and <10 are not reported to preserve anonymity.

© 2025 Wallace ME et al. *JAMA Network Open.*
