# Supplementary material for: Pregnancy-Associated Mortality Due to Homicide, Suicide, and Drug Overdose
Source: JAMA Netw Open. 2025 Feb 11;8(2):e2459342. doi: 10.1001/jamanetworkopen.2024.59342 (PMC11815526; doi:10.1001/jamanetworkopen.2024.59342)
Supplement: Supplement 2. — Data Sharing Statement [file jamanetwopen-e2459342-s002.pdf]

# Data Sharing Statement

Wallace. Pregnancy-Associated Mortality in the US. *JAMA Netw Open*. Published February 11, 2025. doi:10.1001/jamanetworkopen.2024.59342

**Data**
**Data available:** No
